# T-Cell Responses to the DBLα-Tag, a Short Semi-Conserved Region of the *Plasmodium falciparum* Membrane Erythrocyte Protein 1

**DOI:** 10.1371/journal.pone.0030095

**Published:** 2012-01-17

**Authors:** Evelyn N. Gitau, James Tuju, Liz Stevenson, Eva Kimani, Henry Karanja, Kevin Marsh, Peter C. Bull, Britta C. Urban

**Affiliations:** 1 KEMRI-Wellcome Trust Collaborative Programme, Centre for Geographic Medicine Coast, Kilifi, Kenya; 2 Liverpool School of Tropical Medicine, Liverpool, United Kingdom; 3 Centre for Tropical Medicine, Nuffield Department of Internal Medicine, Oxford University, Oxford, United Kingdom; University of Copenhagen, Denmark

## Abstract

The *Plasmodium falciparum* erythrocyte membrane protein 1 (PfEMP1) is a variant surface antigen expressed on mature forms of infected erythrocytes. It is considered an important target of naturally acquired immunity. Despite its extreme sequence heterogeneity, variants of PfEMP1 can be stratified into distinct groups. Group A PfEMP1 have been independently associated with low host immunity and severe disease in several studies and are now of potential interest as vaccine candidates. Although antigen-specific antibodies are considered the main effector mechanism in immunity to malaria, the induction of efficient and long-lasting antibody responses requires CD4+ T-cell help. To date, very little is known about CD4+ T-cell responses to PfEMP1 expressed on clinical isolates. The DBLα-tag is a small region from the DBLα-domain of PfEMP1 that can be amplified with universal primers and is accessible in clinical parasite isolates. We identified the dominant expressed PfEMP1 in 41 individual clinical parasite isolates and expressed the corresponding DBLα-tag as recombinant antigen. Individual DBLα-tags were then used to activate CD4+ T-cells from acute and convalescent blood samples in children who were infected with the respective clinical parasite isolate. Here we show that CD4+ T-cell responses to the homologous DBLα-tag were induced in almost all children during acute malaria and maintained in some for 4 months. Children infected with parasites that dominantly expressed group A-like PfEMP1 were more likely to maintain antigen-specific IFNγ-producing CD4+ T-cells than children infected with parasites dominantly expressing other PfEMP1. These results suggest that group A-like PfEMP1 may induce long-lasting effector memory T-cells that might be able to provide rapid help to variant-specific B cells. Furthermore, a number of children induced CD4+ T-cell responses to heterologous DBLα-tags, suggesting that CD4+ T-cells may recognise shared epitopes between several DBLα-tags.

## Introduction

Clinical immunity to malaria is achieved only after repeated infection with *Plasmodium falciparum* asexual bloodstage parasites. The *Plasmodium falciparum* erythrocyte membrane protein 1 (PfEMP1) mediates adhesion of mature forms of infected erythrocytes to endothelial cells and is central to pathogenesis and protective immune responses and also involved in immune evasion. (reviewed in [1]). Variants of PfEMP1 are encoded by approximately 60 var genes per haploid genome which undergo clonal antigenic variation and are extremely diverse both within and between parasite isolates [2]. In children living in malaria- endemic areas, repeated exposure to a wide range of different PfEMP1 expressed on parasite isolates results in the acquisition of a repertoire of antibodies against different variants that is associated with protection [3], [4]. Furthermore, parasite isolates from children suffering from severe malaria or non-immune children were more likely to be recognised by sera from semi-immune children suggesting that parasites from non-immune children and those with severe disease express antigenically restricted repertoires of PfEMP1 [5], [6].

With the whole genome sequence of the laboratory isolate 3D7 it became evident that *var* genes encoding PfEMP1 can be grouped into three major types, Group A, B and C, suggesting their stratification into separate and potentially functionally distinct groups [7], [8], [9]. This basic concept has now been confirmed using sequence information from additional clinical and laboratory isolates [10]. We (Bull and colleagues) developed a sequence classification system based on a small semi-conserved area of the DBLα-domain of PfEMP-1, the DBLα-tag, which allows classification of the entire *var* gene repertoire of clinical isolates. The amino acid sequence of amplified DBLα-tags can be grouped according to the number of cysteine (cys2 or cys4), the presence of sequence signatures at “Positions of Limited Variation” (PoLV) and through sharing of a limited number of sequence blocks within the hypervariable regions [11], [12]. A schematic diagram of the DBLα-tag is shown in the Figure S1. Using this system we showed differential expression of distinct subgroups of *var* genes in parasite isolates from children suffering from different syndromes of severe disease and in non-immune children [11], [13]. Importantly, a subgroup of DBLα-tags that share blocks of diverse sequence overlap with group A *var* genes identified in the 3D7 genome [12] and are independently associated with young host age and severe malarial syndromes [3]. Although likely, whether serologically and genetically defined subgroups of PfEMP1 identify the same group of variants has not yet been determined.

T-cells play a critical role in protection, not only by providing help to B cells but also through the secretion of cytokines that activate macrophages and may have parasiticidal activity (reviewed in [14]. Rodent models of malaria have repeatedly shown that T-cells are essential for early control of parasitaemia and that elimination of parasites is dependent on both T and B-cells [15], [16]. To date, only a few studies have analysed T-cell responses to PfEMP1 using either recombinant domains or peptides based on a very limited number of PfEMP1 molecules identified in laboratory isolates. These studies showed that a proportion of malaria-exposed children and adults responded to DBLα or exon2 by proliferation or cytokine secretion in an antigen-specific manner [17], [18]. In both studies, proliferation of CD4+ T-cells in response to antigen was observed in a small number of unexposed European blood donors after seven days of culture. Under these conditions, priming of naive T-cells occurs in culture and it can not be determined whether the apparent CD4+ T-cell response of unexposed European blood donors was non-specific or an antigen-specific response of naive CD4+ T-cells [19]. By contrast, CD4+ T-cell responses to one CIDRα domain were observed in both malaria-exposed and unexposed individuals but only the response in malaria-exposed individuals was MHC class II restricted [17], [20]. However, the use of recombinant domains representing PfEMP1 expressed on laboratory isolates as described in these studies may not capture T-cell responses specific for PfEMP1 expressed on parasite(s) circulating within a population and therefore miss a significant proportion of responses.

We were interested in whether there are differences in the type, magnitude or duration of CD4+ T-cell responses to PfEMP1 dominantly expressed at the time of acute disease in children suffering from severe malaria or infected with parasites expressing specific subgroups of PfEMP1. We expressed recombinant DBLα-tags from parasites isolated from clinical cases of malaria and determined CD4+ T-cell responses in those children who provided the clinical parasite isolate at the time of acute disease and during convalescence. Although the DBLα-tag is only a small part of the entire PfEMP1 molecule, it can serve as a model antigen to determine whether a specific subset of PfEMP1 is associated with a particular type of immune response.

Here we show that the magnitude, duration and type of DBLα-tag specific CD4+ T-cells responses were not different between children with severe or mild malarial symptoms. However, a small group of children infected with parasites expressing group A variants of PfEMP1 were more likely to induce antigen-specific IFNγ-secreting CD4+ T-cells 16 weeks after follow-up indicating that the quality of T-cell responses to group A PfEMP1 differs from those induced against the more diverse non-GroupA PfEMP1. Surprisingly, we observed CD4+ T-cell responses to heterologous DBLα-tags (DBLα-tags from a clinical isolate a child was not infected with) in a sub-group of children tested despite the extreme sequence diversity of DBLα-tags. These data suggested that DBLα-tags contain a number of shared T-cell epitopes that may be recognised by cross-reactive CD4 T-cells.

## Results

### Phenotype of T-cell responses to PfEMP1 during acute disease and follow-up

**Figure 1:**
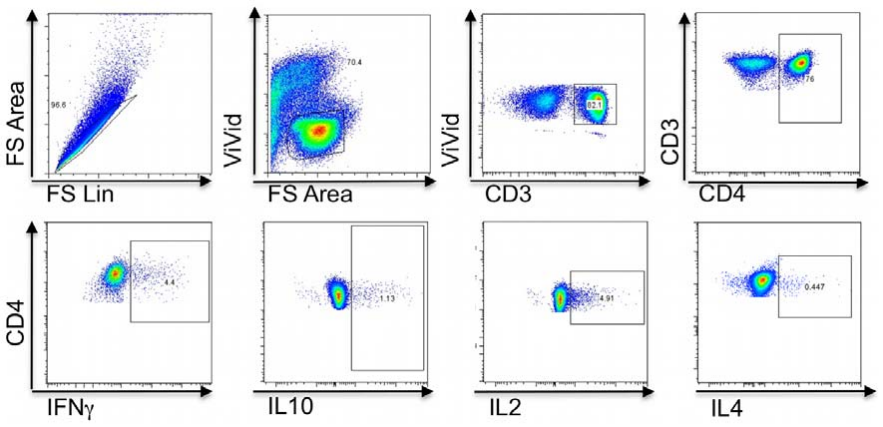
Gating Strategy used to identify T-cell responses. PBMCs were gated to eliminate doublets and dead. CD3+lymphocytes were identified and gated for CD4+ and CD8+ T-cells. Within each T-cell subset, the proportion of IFNγ, IL10, IL2, and IL4 producing cells were determined. IFNγ+IL10+ and IL2+IL4+ double producers were determined by Boolean gating.

The main aim of this study was to analyse the magnitude, duration and phenotype of CD4+ T-cell responses to the PfEMP1 variant expressed in parasites infecting individual children during acute disease and convalescence. PBMCs collected at presentation, 4 weeks and 16 weeks after the acute attack were stimulated with the DBLα-tag (EMBL accession nos. FR874861-FR874900, HE611335) representing the dominant PfEMP1 expressed on the parasites a given child was infected with at the time of acute disease and determined the proportion of DBLα-tag-specific CD4 T-cells producing either IFNγ, IL10, IL2 or IL4 (Figure 1). We used the expression homologous DBLα-tags to identify CD4+ T-cell responses to the DBLα-tag derived from the clinical isolate a given child was infected with and heterologous DBLα-tags to identify CD4+ T-cell responses to DBLα-tags derived from clinical isolates of other children. Baseline haematological parameters are described in Table 1.

**Table 1:** Baseline characteristics of study population.

	Acute episode	4 weeks follow up	16 weeks follow up
**age** (months)	42.1 (25.6–57.4)	43.1 (26.6–58.4)	46.1 (29.6–61.4)
**parasite density** (10^3^/µl)	190 (100–369)	0^a^	0^b^
**WBC** (10^6^/µl)	8.4 (6.6–13.7)	8.2 (6.4–12.4)	9.1 (7–10.3)
**RBC** (10^9^/µl)	4 (3.5–4.5)	4.8 (4.2–5.4)*	4.8 (4.2–5.1)*
**Hb** (g/dl)	9.2 (7.4–10.2)	10.6 (9.5–12)*	10.7 (9.4–11.9)*

Shown are median and in parenthesis 25^th^ and 75^th^ percentile.

*p≤0.05 compared to acute disease, Wilcoxon signed rank test.

a one child had a parasite density of 514/µl blood 4 weeks after the acute event.

b one child had a parasite density of 138×10^3^/µl blood 16 weeks after the acute event.

**Figure 2:**
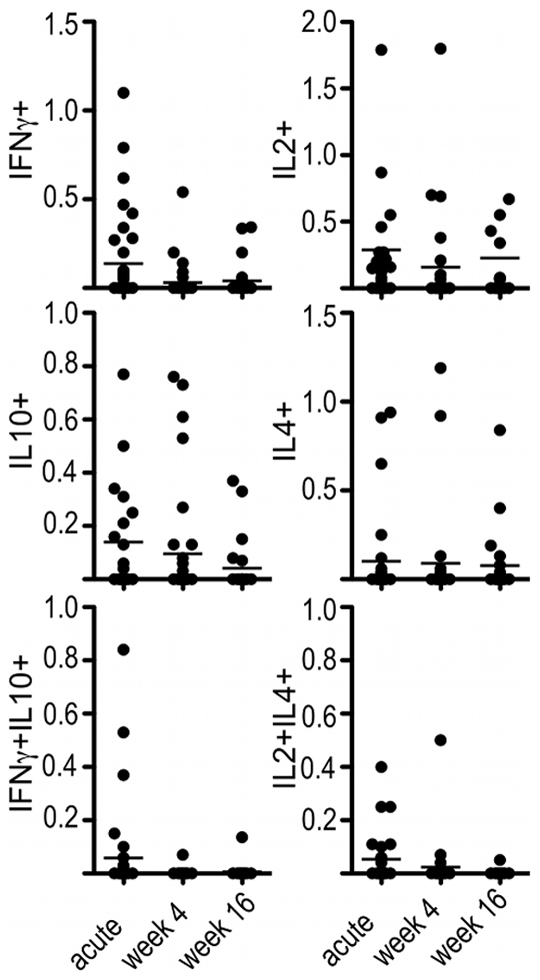
Percentage of CD4+ T-cells producing cytokines after stimulation with the homologous DBLα-tag. PBMCs from children obtained during acute malaria, 4 weeks and 16 weeks after the acute event were stimulated with recombinant DBLα-tag representing the dominant expressed PfEMP1 a given child was infected with. CD4+ T-cells were stained for production of INFγ and IL10 or IL2 and IL4 by intracellular cytokine staining. Shown are dot plots of percentages of CD4+ T-cells producing the indicated cytokine or cytokine combination during acute malaria or 4 and 16 weeks after the acute episode. Horizontal lines indicate the median.

The majority of children (85%, n = 35) induced CD4+ T-cells producing at least one of the tested cytokines IL2, IL4, IL10 and IFNγ in response to activation with the homologous DBLα-tag during acute disease (Figure 2). Antigen-specific, cytokine producing CD4+ T-cells were detected in 58% (n = 24) and 46% (n = 20) of children 4 weeks and 16 weeks after the acute malaria attack, respectively. The proportion of antigen-specific, cytokine-producing CD4+ T-cells was comparable between different time points (Mann Whitney U test, p>0.05) although the number of children mounting a CD4+ T-cell response to the homologous DBLα-tag dropped significantly for CD4+IFNγ+, CD4+IFNγ+IL10+ and CD4+IL2+IL4+ T-cells over the 4 months following the acute episode (Pearson's χ^2^ for trend, p<0.05; Table 2).

**Table 2:** Magnitude of cytokine secretion by CD4+ T-cells after stimulation with DBLα-tags.

		Acute episode	4 weeks follow up	16 weeks follow up
**IFNγ+**	Median all	0.017 (0–0.18)	0 (0–0.02)	0 (0–0.025)
	N (%) responders*	15 (36.5%)	6 (14.6%)	5 (12.2%)
	Median responders	0.27 (0.08–0.42)	0.16 (0.09–0.54)	0.2 (0.06–0.34)
**IL10+**	Median all	0 (0–0.11)	0 (0–0.06)	0 (0–0.005)
	N (%) responders	11 (26.8%)	10 (24.3%)	5 (12.2%)
	Median responders	0.25 (0.14–0.42)	0.2 (0.08–0.61)	0.15 (0.08–0.33)
**IFNγ+IL10+**	Median all	0 (0–0.007)	0 (0–0)	0 (0–0)
	N (%) responders*	7 (17%)	1 (2.4%)	1 (2.4%)
	Median responders	0.15 (0.08–0.61)	na	na
**IL2+**	Median all	0.06 (0–0.27)	0 (0–0.1)	0.008 (0–0.27)
	N (%) responders	15 (36.5%)	9 (22%)	9 (22%)
	Median responders	0.22 (0.14–0.5)	0.21 (0.1–0.69)	0.34 (0.07–0.55)
**IL4+**	Median all	0 (0–0.038)	0 (0–0.025)	0 (0–0.12)
	N (%) responders*	8 (19.5%)	5 (12.2%)	7 (17%)
	Median responders	0.18 (0.05–0.78)	0.13 (0.06–0.92)	0.19 (0.1–0.62)
**IL2+IL4+**	Median all	0.004 (0–0.008)	0 (0–0.008)	0.002 (0–0.011)
	N (%) responders	11 (26.8%)	3 (7.3%)	2 (4.8%)
	Median responders	0.1 (0.05–0.18)	0.06 (0.05–0.28)	0.04, 1.7

*p≤0.05, Pearson's χ^2^ for reduction in the number of responders over time.

**Figure 3:**
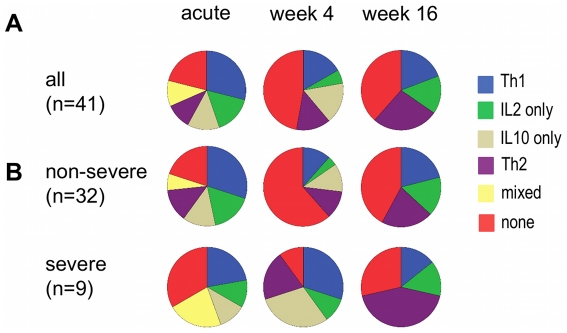
Phenotype of CD4+ T-cells stimulated with the homologous DBLα-tag. The phenotype of CD4+ T-cell in response to the homologous DBLα-tag was classified for each child and time point as followed: Th1, secretion of IFNγ alone or together with IL2 and/or IFNγ+IL10+; Th2, secretion of IL4 alone or in the presence of CD4+ T-cells secreting IL2 or IL10; IL2 alone: detection of only IL2-producing CD4+ T-cells; IL10 alone: detection of only IL10-producing CD4+ T-cells; mixed: detection of a mixed profile such as IFNγ-producing CD4+ T-cells together with IL4- or IL10-producing CD4+ T-cells or IL2- and IL10-producing CD4+ T-cells; none: children who did not induce any antigen-specific CD4+ T-cells. Shown are pie charts of the proportion of all children (A) with a particular profile or (B) grouped by disease severity.

To describe changes in the type of CD4+ T-cell responses over time, we classified the CD4+ T-cell response to homologous DBLα-tags as a Th1 or Th2 response, CD4+IL2+ or CD4+IL10+ only response, a mixed response, no response. During acute disease, CD4+ T-cells from 11 children (27%) produced IFNγ either alone or together with IL2 (n = 8) indicative of a Th1 response. This profile was maintained in 3 children for 4 weeks and in only 1 child for 16 weeks whereas 6 children showed a Th1 profile during the follow-up period but not during acute disease (Figure 3A). We detected a Th2 profile (defined by secretion of IL4 either alone (n = 4) or together with IL2 (n = 4) and/or IL10 (n = 3) in the absence of any CD4+IFNγ+ T-cells) in 4 children at acute disease but only one child maintained this profile over the entire period of 16 weeks. By contrast, 4 and 6 children gained CD4+IL4+ T-cell responses to the DBLα-tag at 4 weeks and 16 weeks, respectively, shifting from either a Th1 or mixed Th1/Th2 response at acute disease to a Th2 response in all but one child.

Antigen-specific, CD4+IL10+ T-cells were detected in 11 children either alone (n = 5) or together with CD4+IFNγ+ T-cells (n = 5). Four weeks after the acute episode, 6 children showed a sole CD4+IL10+ T-cell response, which was maintained in 3 children or switched from a CD4+IFNγ+ T-cell response either alone or mixed with CD4+IL10+ T-cells (n = 3). We detected IFNγ+IL10+ double producing CD4+ T-cells in seven children (n = 7) but only in combination with either CD4+IL10+ or CD4+IFNγ+ T-cells. With the exception of one child, DBLα-tag specific CD4+IFNγ+IL10+ T-cells were observed only at the time of acute disease. Although not significant, children who mounted DBLα-tag specific IFNγ+IL10+ double producing CD4+ T-cells tended to have a higher parasitaemia at acute disease (median (25^th^ and 75^th^ interquartile) 255,680 parasites/ml blood (191,855–337,230) in children with IFNγ+IL10+ double producing CD4+ T-cells and 148,240 parasites/ml blood (65,080–256,470) in those without; Mann Whitney p = 0.08).

A small proportion of children displayed only CD4+IL2+ T-cells in response to the homologous DBLα-tag at acute disease (n = 6) indicating that these T-cells were primed but not yet fully differentiated. IL2-producing CD4+ T-cell responses in the absence of any other cytokine-producing T-cells were also observed during follow-up but only in those children that either induced no CD4 T-cell response or a Th1 response during acute disease.

Together these results indicate that during acute malaria disease, CD4+ T-cell responses are dominated by a Th1 response sometimes in combination with IL10-producing CD4+ T-cells. By contrast, CD4+ T-cell responses 4 weeks and 16 weeks following the acute attack showed a more focused profile in those children that maintained a DBLα-tag specific response.

### CD4+ T-cell responses to DBLα-tags in children with severe disease

We wondered whether children suffering from severe, life-threatening malaria differed with respect to the magnitude, duration or type of CD4+ T-cells responses specific for the homologous DBLα-tag from children suffering from moderate malaria. Children with severe malaria were similar with respect to age, WBC and RBC count or Hb concentration to children with mild to moderate malaria. There were no differences in either the number of responders or the magnitude of cytokines produced by CD4+T-cells in response to homologous DBLα-tags between children suffering from severe or mild malaria either during acute disease or over the 4 months follow-up period. Likewise, there was no difference in the overall DBLα-tag specific T-cell profiles between children with severe disease and mild disease (Figure 3B).

### T-cell responses to different subsets of DBLα-tag

**Figure 4:**
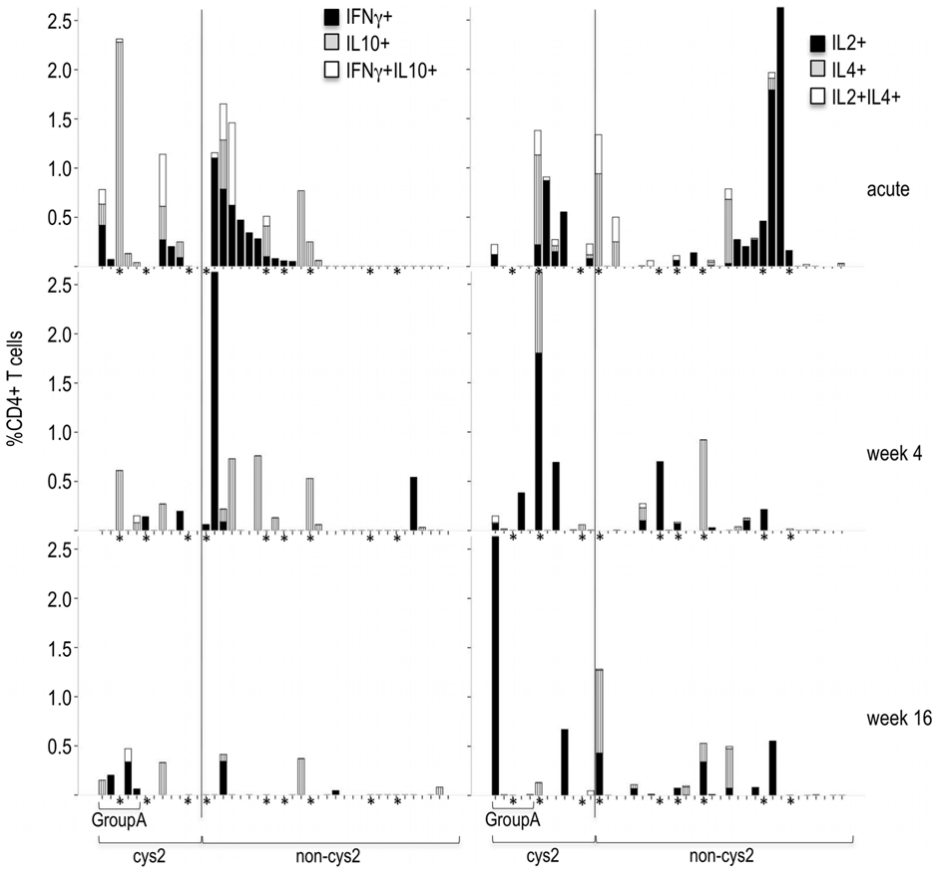
Cumulative percentage of cytokine producing CD4+ T-cells after stimulation with homologous DBLα-tag by PfEMP1 subgroup. Shown are cumulative bar-graphs of CD4+ T-cells producing cytokines indicated for each child. Data are grouped by the PfEMP1 subclass of the homologous DBLα-tag at acute disease, week 4 and week 16 after the acute event. Children suffering from severe disease are indicated by a star. Note that the order of children is the same in each plot.

Expression of cys2 PfEMP1 and within these, the subgroup of group A-like PfEMP1, had been associated with severe disease and with low levels of existing immunity in young children [3]. We therefore determined the *var* gene classification of each recombinant DBLα-tag and compared CD4+ T-cell responses in children infected with parasites that expressed one dominant cys-2 PfEMP1 (n = 12) or Group A-like PfEMP1 (n = 5, a subgroup of cys2 sequences) with those that did not. As with severe disease, these children did not differ with respect to age, WBC, RBC counts or Hb. Cys2 PfEMP1 were expressed on clinical isolates from three children suffering from severe malaria and nine children suffering from non-severe malaria. However, children infected with parasites that expressed one dominant cys2 PfEMP1 showed a higher proportion of DBLα-tag specific CD4+IL10+ T-cells (median (25^th^ and 75^th^ percentile): 0.13 (0–0.28) for cys2 DBLα-tags; 0 (0–0) for non-cys2 DBLα-tags, Mann Whitney U test p = 0.015) and a higher frequency of responders (6/9 responders for cys2; 5/27 responders for non cys2, Pearson's χ^2^ = 7.4, df = 1, p = 0.007) during acute disease than children infected with parasites that expressed a dominant non-cys2 PfEMP1 (Figure 4). Conversely, the phenotype of the CD4+ T-cell response in children infected with parasites that expressed a dominant cys2 PfEMP1 at acute disease changed to a predominantly IFNγ+ response, both with respect to the number of children (3/6 responders for cys2; 2/18 responders for non cys2, Pearson's χ^2^ = 4.1, df = 1, p = 0.042) and the magnitude of the response (median % of CD4+ T-cells (25^th^ and 75^th^ percentile): 0.03 (0–0.23) for cys2 DBLα-tags; 0 (0–0) for non-cys2 DBLα-tags, Mann Whitney U test p = 0.049) 16 weeks after the acute episode. However, this trend was driven by a subgroup of children infected with parasites that expressed a dominant Group A-like PfEMP1 at acute disease (median % of CD4+ T-cells (25^th^ and 75th percentile): 0.13 (0.05–0.3) for group A DBLα-tags; 0 (0–0) for non-group A DBLα-tags, Mann Whitney U test p = 0.005; 3/5 responders for group A; 2/20 responders for non group A, Pearson's χ^2^ = 8.5, df = 1, p = 0.003). In summary, cys2 DBLα-tags induced CD4+ T-cell responses that differed in phenotype and magnitude from CD4+ T-cell responses induced by non-cys DBLα-tags suggesting that parasites that express a dominant cys2 PfEMP1 induced a different CD4+ T-cell response compared to parasites that express a dominant cys2 PfEMP1.

### T-cell responses to heterologous DBLα-tags

**Figure 5:**
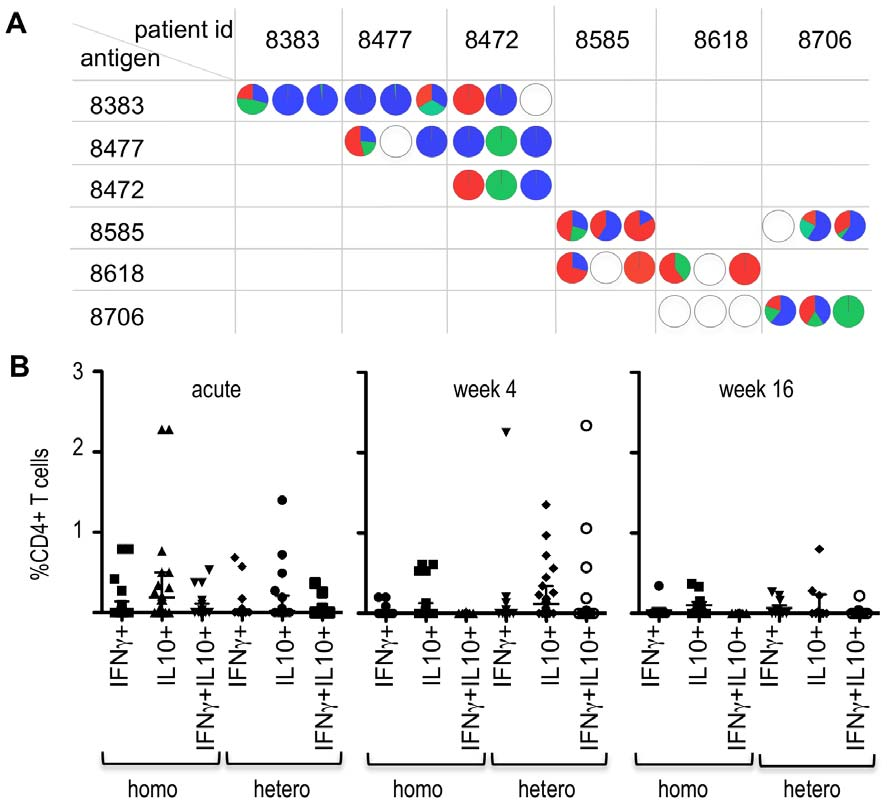
CD4+ T-cells responses response to homologous or heterologous DBLα-tags. (A) Representative examples of CD4+ T-cell responses to homologous and heterologous DBLα-tags for 6 children. Pie charts indicate the IFNγ+ (red), IL10+ (blue) and IFNγ+IL10+ double producing (green) CD4 T-cells as a proportion of the total cytokine producing CD4+ T-cells. White circles indicate the absence of a response. Within each cells, the sequence of circles indicates the proportion of CD4+ T-cells at acute disease (left), and 4 weeks (middle) and 16 weeks (right) after the acute episode. (B) Dot plots of the percentage of CD4+ T-cell responses to homologous (homo) and heterologous (hetero) DBLα-tags during acute malaria or 4 and 16 weeks after the acute attack. Horizontal lines indicate the median.

For some children we had sufficient number of PBMCs to evaluate CD4+ T-cell responses to another, heterologous DBLα-tag. The heterologous DBLα-tag tested was chosen randomly from a child tested at the same day. Overall, we analysed heterologous responses in 14 children during acute disease, in 16 children 4 weeks and in 10 children 16 weeks after the acute malaria episode. In total, CD4+ T-cell responses to 25 pairs of homologous and heterologous DBLα-tag were compared at one or more time points. The pairwise identity between homologous and heterologous DBLα-tags ranged from 33.6% to 56.7% (Table S1). For only four pairs, both the homologous and the heterologous DBLα-tag were classified as cys2 PfEMP1. CD4+ T-cell responses to heterologous DBLα-tags varied between children, with some children inducing no CD4+ T-cell response, a similar CD4+ T-cell response profile or a different CD4+ T-cell response profile to the heterologous DBLα-tags compared to the homologous DBLα-tag (Figure 5A). There was no obvious tendency for either a similar or different type of CD4+ T-cell response to the heterologous DBLα-tag compared to the homologous DBLα-tag (Figure 5B). These data suggest that some children had been exposed to T-cell epitopes similar to those observed in the heterologous DBLα-tag before.

**Figure 6:**
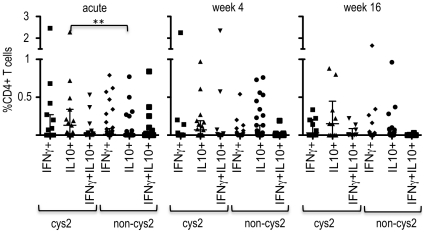
CD4+ T-cells responses response to cys2 and non-cys2 DBLα-tags. Dot plots of the percentage of CD4+ T-cell responses to cys2 and non-cys2 DBLα-tags whether or not they represent homologous or heterologous DBLα-tags during acute malaria or 4 and 16 weeks after the acute attack. Horizontal lines indicate the median.

When we considered CD4+ T-cell responses to cys2 DBLα-tags and non-cys2 DBLα-tags for the homologous and heterologous DBLα-tags together, we observed a similar trend with respect to the differences in the type duration and magnitude of CD4+ T-cell responses. Overall, the magnitude of CD4+ T-cells responses and the proportion of responders was higher when PBMCs were stimulated with cys2 DBLα-tags or group A DBLα-tags compared to stimulation with non-cys2 DBLα-tags (Figure 6). Comparable to CD4+ T-cell responses to the homologous DBLα-tag, a higher percentage of CD4+IL10+ T-cells (median % of CD4+ T-cells (25^th^ and 75^th^ percentile): 0.13 (0–0.34) for cys2 DBLα-tags; 0 (0–0) for non-cys2 DBLα-tags, Mann Whitney U test p = 0.003) and a higher proportion of responders (10/15 responders for cys2; 8/38 responders for non cys2, Pearson's χ^2^ = 9.9, df = 1, p = 0.002) was detected during acute disease whereas CD4+IFNγ positive T-cells dominated 16 weeks after the acute attack with respect to the number of responders (5/10 responders for cys2; 5/31 responders for non cys2, Pearson's χ^2^ = 4.7, df = 1, p = 0.03). Likewise, the number of responders inducing CD4+IL10+ T-cells after stimulation with group A-like DBLα-tags was higher compared to other DBLα-tags during acute disease (5/7 responders for group A; 13/46 responders for non-group A, Pearson's χ^2^ = 5.05, df = 1, p = 0.025) whereas induction of CD4+IFNγ+ T-cells in response to group A DBLα-tags dominated 16 weeks after the acute attack (4/6 responders for group A; 6/35 responders for non group-A, Pearson's χ^2^ = 6.8, df = 1, p = 0.009). Together, these data indicate that cys2 PfEMP1, and particularly group A PfEMP1, may induce a different T-cell response than non-cys2 PfEMP1.

## Discussion

Here we report that DBLα-tag-specific T-cell responses are readily detected in children with acute malaria and can be identified 16 weeks after the acute episode in a proportion of children indicating that some of them may induce long-lasting effector T-cells. Importantly, the DBLα-tag used as antigen here was identified as the dominant PfEMP1 expressed on the clinical parasite isolate circulating in a given child and thus we measured T-cell responses to the individual PfEMP1 variant a child was actually exposed to.

Obviously, our approach has limitations. First, the entire PfEMP1 molecule will harbour many more T-cell epitopes and some of those lying outside the DBLα-tag may be more dominant. Second, we assumed that the T-cell phenotype(s) we detected using the DBLα-tag as an antigen will reflect the T-cell phenotype induced by the entire PfEMP1 molecule since activation of T-cell recognizing different epitopes of one PfEMP1 variant will occur in a similar environment with respect to co-stimulation and cytokines provided by antigen presenting cells. However, different parts of a given molecule may be processed at different rates and thus may affect epitope density or delays in T-cell priming by dendritic cells with consequences for T-cell function [21]. Bearing these assumptions in mind, we nevertheless made significant observations. First, we detected DBLα-tag specific IFNγ and IL10+ double-producing CD4+ T-cells in a proportion of children. Second, CD4+ T-cell responses to cys2 DBLα-tag showed a distinct profile with IL10 secretion during acute disease but IFNγ secretion 16 weeks after the acute event. Finally, we detected CD4+ T-cell responses to heterologous DBLα-tags despite considerable sequence diversity between DBLα-tags suggesting that at least some T-cell responses may be cross-reactive.

The majority of children responded to the homologous PfEMP1 by induction of CD4+IFNγ and CD4+IL2+ producing T-cells, indicating a Th1 profile either alone or in combination with IL10-secreting CD4+ T-cells. In addition, we detected IFNγ and IL10+ double-producing CD4+ T-cells, which have recently been described [22]. These T-cells reflect a normal differentiation process in Th1 responses providing a negative feedback loop in the presence of high antigen load or high concentrations of IL12 and are thought to regulate an otherwise dangerous inflammatory response. Most IL10-producing Th1 T-cells co-produce IFNγ but sole production of IL10 after repeated stimulation has been reported [23], [24], [25], [26], [27]. Importantly, IL10-producing Th1 cells switch to IFNγ production after a period of rest and some authors now suggest that at least some of the IL10-producing Tr1 cells may present late stages of Th1 [28]. In agreement with these studies, children with IL10-producing Th1 T-cells tended to have high parasitaemia during acute disease. IL10-producing Th1 T-cells had first been described over two decades ago but to our knowledge this is the first report of these cells in malaria [29].

When we analysed CD4+ T-cell responses by PfEMP1 subgroup, we observed that children infected with parasites expressing a cys2 PfEMP1 tended to mount IL10+ CD4+ T-cells during acute disease but displayed IFNγ+ CD4+ T-cell responses 16 weeks after the acute episode, the later was driven by group A-like DBLα-tags. When we considered all cys2 DBLα-tags tested independent of whether they reflected heterologous or homologous responses, we noted a similar trend to that observed with homologous cys2 DBLα-tag. The shift from a T-cell response dominated by IL10 at acute disease to one dominated by IFNγ may be the result of a regulatory response to inflammation either due to normal differentiation of Th1 T-cells or due to induction of IL-10-producing regulatory T-cells [22], [28]. During acute disease, high antigen-load and pro-inflammatory conditions may favour switching of Th1 cells to the production of IL10 [28]. Alternatively, antigen-specific Th1 T-cells may redistribute to the spleen during acute disease and re-enter the circulation only after the infection has been cleared [30]. To differentiate between these options, both IL10- and IFNγ-producing DBLα-tag specific T-cells require further characterisation. Nevertheless, pending confirmation in a larger group of children, our results indicate that cys2 DBLα-tags and within this subset, group A-like DBLα-tags may induce a stable Th1 effector memory response that may contribute to the rapid acquisition of immunity to this particular subset of PfEMP1 variants. Although we can not exclude the possibility that antigen-specific T-cell responses detected 4 and 16 weeks after the acute episode were due to additional infections, it seems unlikely because all children were tested for presence of parasites by microscopy at the clinical examination during follow-up visits and they occurred mainly during the dry season when transmission is low. Stable populations of effector memory T-cells have been reported in humans in a rodent model of malaria [31], [32]. Both cys2 and Group A PfEMP1 variants are frequently expressed in non-immune hosts such as young children but also in children suffering from severe malarial disease [3]. Thus, memory CD4+ T-cells specific for cys2 and Group A PfEMP1 may be induced during the first few infections and if they were expressed early in later infections but maybe not maintained due to negative immune-selection, adaptive immune responses against group A PfEMP1 variants might be boosted resulting in the maintenance of effector memory T-cells.

We were able to test CD4+ T-cell responses to heterologous DBLα-tags in a small subset of children. Overall, heterologous responses varied both between antigens and between patients with no obvious trend towards reduced or increased antigen-specific CD4+ T-cell responses or the proportion of responders. CD4+ T-cell responses to heterologous DBLα-tags probably reflect previous or concurrent exposure of individual children to PfEMP1 variants with similar DBLα-domains either during the current or a previous infection. In all clinical isolates, more then one DBLα-tag sequence was detected probably representing expression of switched PfEMP1 or infection with additional parasite clones. Thus, all children in this study were exposed to more then one PfEMP1 variant over the course of acute infection and could have mounted an immune response to various DBLα-tag. However, over its entire length, different DBLα-tags and indeed DBLα domains show only limited identity and a large study on more than 14,000 DBLα-tag sequences in Kilifi, Kenya observed only a small number of identical sequences [13]. Therefore, chance exposure to the PfEMP1 represented by the heterologous DBLα-tag seems highly unlikely. By contrast, this observation suggests that the DBLα-tag region contains T-cell epitopes that are relatively conserved and shared between several DBLα domains. Indeed, a study by Sanni et al. suggested that targets of CD4+ T-cell responses to PfEMP1 are relatively conserved because they detected CD4+ T-cell responses to pools of peptides derived from semi-conserved blocks of 15 variants of PfEMP1 [18]. Our own preliminary analysis of predicted T-cell epitopes within the 41 DBLα-tags showed that predicted high affinity peptides often span semi-conserved areas. For instance the region between PoLV3 and PoLV4 shows distinct sequence signatures (also known as homology blocks) that differentiate between cys2 and cys4 DBLα-tags [10]. In our preliminary analysis we observed a higher proportion of peptides predicted to bind to MHC class II alleles in cys2 DBLα-tags than in cys4 DBLα-tags. Cross-recognition of peptides from a subset of antigenically restricted PfEMP1 within MHC class II supertypes would allow rapid activation of memory T-cells that can provide help to B-cells specific for the cognate antigen and may result in earlier and more efficient antibody responses. CD4+ T-cell epitopes may not be subjected to the same degree of immune selection as B-cell or CD8 T-cell epitopes because CD4+ T-cell effector function is often indirect through activation of other cell types, their frequency is generally lower than that of CD8+ T-cells and protection conferred by CD4+ T-cells in other infections usually limits disease severity but does not induce sterile protection [33], [34].

In summary, our data suggest that DBLα-tags from group A PfEMP1 may contain a limited set of T-cell epitopes that are shared between variants of group A DBLα-tags. If this were the case, subsequent encounter of group A PfEMP1 might activate cross-reactive CD4 T-cells that can provide rapid help to B cells. This hypothesis is testable using a combination of bioinformatic approaches and cellular assays. If it can be confirmed, the design of vaccines targeting PfEMP1 variants associated with severe, life threatening illness may become feasible.

## Methods

### Ethics Statement

The study was approved by the Kenyan National Ethics Review Committee (protocol no. 1131) and the Oxford Tropical Research Ethics Committee (protocol no. 30-06). Parents or Guardians of the children provided written informed consent.

### Study cohort

We analysed blood samples from 41 children with acute malaria attending Kilifi District Hospital between 2006 and 2009. After parents or guardians provided informed consent for participation of their children in the study, children donated a venous blood sample of 3 ml at presentation with acute malaria, and 4 weeks and 4 months after the acute episode. Children who were admitted with impaired consciousness (Blantyre Coma Score <5), severe anaemia (Hb <5 g/dl and at least 10,000 parasites/µl blood) or severe respiratory distress (deep breathing and chest recession) were classified as suffering from severe malaria (n = 9). During follow-up visits, children were clinically examined, tested for the presence of *Plasmodium* species by microscopy and received a differential blood count. Children who were acutely ill received medical treatment as required. Parents or Guardians of the children provided informed consent.

### Processing of blood samples

Heparinised blood was spun at 2000 rpm, plasma removed, aliquoted and stored at −80°C. The remaining blood cells were diluted to a total volume of 5 ml in RPMI before separation of peripheral blood mononuclear cells (PBMC) with lymphoprep. PBMC were washed twice with RPMI, resuspended in 10%DMSO/FCS and stored in liquid nitrogen. The remaining red blood cells (RBCs) were washed twice in RPMI and RBCs and granulocytes were separated by plasmagel flotation. 100 µl of packed RBCs were resuspended in 800 µl Trizol and stored at −80°C for extraction of RNA.

### Isolation and expression of dominant expressed DBLα tags

Dominant expressed PfEMP1 were identified in 41 clinical isolates by the method described in detail elsewhere [11] with the difference that we identified, classified and expressed the single transcript that was most abundant. In brief, RNA was extracted from RBCs, converted to cDNA and amplified using DBLαAF′ (5′-GCACG(A/C)AGTTT(C*/T)GC-3′) and DBLαBR (5′-GCCCATTC(G/C)TCGAACCA-3′) primers targeting a semi-conserved region of the DBLα-domain. PCR-products were cloned into pCR2.1 cloning vector, plasmid extracted from 10–20 colonies and sequenced on an Applied Biosystems 3730 sequencer using Big Dye Terminator v.3.1 cycle sequencing. The dominant DBLα-tag sequence was identified (range 10%–85% of sequenced transcripts), amplified from a representative plasmid by PCR using the DBLαAF′ primer and a DBLαBR primer containing a 3′-stop codon (5′-TTAGCCCATTC(G/C)TCGAACCA-3′). PCR products were ligated into the pEXP5(NT) TOPO vector (Invitrogen) containing and N-terminal His-tag sequence, transformed into TOP10 cells and the insert confirmed by sequencing. The DBLα-tag-containing plasmids were transformed in BL21 DE3pLysS *E. coli* (Invitrogen). Individual colonies were grown to an OD_600_ of 0.4, protein expression induced with 1 mM IPTG, and cells grown for another 4 hours. The cell pellet was harvested by centrifugation and lysed with Bugbuster NT (Novagen) in the presence of Benzonase (Novagen). All DBLα-tags formed inclusion bodies and were purified under denaturing condition on Probond Nickel-chelating resin (Invitrogen) according to manufacturers recommendation. Purified proteins were dialysed over 24 hours against 20 mM TrisHCl, 50 mM NaCl, 6 M Urea pH 4.5 with stepwise reduction of the Urea concentration. Final dialysis was against 20 mM TrisHCl, 50 mM NaCl pH 4.5 for 12 hours. Contaminating endotoxin was removed using Endotrap blue (Hyglos GmbH) according to manufacturers recommendation and the residual endotoxin concentration was determined using the Limulus amoebae-lysate assay (Cambrex Bioscience) according to manufacturers recommendation. The residual endotoxin concentrations ranged from 0.0004–0.003 U/µg protein. The final protein concentration was determined using the BCA assay (Pierce). Recombinant proteins were sterile-filtered with 0.2 µm syringe filters and stored at −80C.

### Intracellular cytokine staining

PBMCs from acute and convalescent samples were thawed and 0.5×10^6^ cells seeded twice in triplicate into 96 well plates in medium (RPMI1698 supplemented with 5% pooled human AB serum, 5 mM glutamine, 10 mM Hepes, 50 µM β-mercaptoethanol, 50 µM kanamycin). Cells were rested overnight before activation with medium alone, 20 µg/ml homologous recombinant DBLα-tag or anti-CD2/CD3/anti-CD28-coated MACSiBead particles (Miltenyi Biotec) in the presence of 1 µg/ml CD28 and CD49d for 2 hours. Cells were incubated for another 18 hours in the presence of brefeldinA. Cells were harvested and stained with ViViD Aqua (Invitrogen) before intracellular cytokine staining was performed as follows: Cells were fixed with Cytofix (Becton Dickinson) for 20 min at RT in the dark, washed twice with Cytoperm (Becton Dickinson) and subsequently stained in Cytoperm with CD3-ECD Beckman Coulter), CD4-PC7 (Beckman Coulter), CD8-APC H7 (Becton Dickinson) and either IFNγ-FITC (R&D) and IL10-PE (Becton Dickinson) or IL2-FITC (R&D) and IL4-PE (Becton Dickinson), for 1 hour at 4°C in the dark. Cells were washed twice, resuspended in Sheath Fluid (Beckmann Coulter) and acquired on a Cyan Analyzer (Beckmann Coulter) within 24 hours. Live, CD3+CD4+ or CD3+CD8+ T-cells were identified and the proportion of IFNγ+IL10-, IFNγ-IL10+ and IFNγ+IL10+ T-cells was determined using FlowJo Africa. Four samples with less than 1000 CD4+ T-cells (n = 2) or failed positive response (cytokine secretion in less than 1% of CD4+ T-cells stimulated with microbeads, n = 2) after staining for CD4+ T-cells secreting IL2, IL4 or both were excluded from further analysis. Values obtained from PBMCs incubated with medium alone were subtracted from values obtained after activation of PBMCs with DBLα-tags. When individual gates of cytokine-secreting CD4+ T-cells had less then 10 positive events or their percentage was below 0.03%, the response was recorded as zero.

### Statistical data analysis

All data were analysed using PAWStatistics version 18. Comparison of continuous variables between different groups was done using Mann-Whitney rank sign test and for comparison of categorical variables the Pearson χ^2^ or Fisher's exact test was used. P-values less than 0.05 were considered significant. For graphic representation of data PAWStatistics version 18.0 and Graphpad Prism version 5 was used.

## Supporting Information

Figure S1 **Schematic Overview of the DBLα domain organisation.** (A) Indicated is the relative position of homology blocks (HB) 1–5 common to all DBL domains. Universal primers amplifying the DBLα-tag target conserved sequences in HB3 and HB2 and are indicated by black arrows. The “Positions of Limited Variation” (PoLV) 1 to 4, which together with the number of cysteine's in each DBLα-tag form the basis of the DBLα-tag classification. (B) Sequence signature of cys2 and cys4 DBLα-tags. PoLV1-4 are indicated by blue brackets.
(TIF)

Table S1 *MUSCLE alignment, shown is the percentage and in parenthesis the number of identical amino acids.
(DOC)
