# Supplementary figures and images for: T-Cell Responses to the DBLα-Tag, a Short Semi-Conserved Region of the Plasmodium falciparum Membrane Erythrocyte Protein 1
Source: PLoS One. 2012 Jan 17;7(1):e30095. doi: 10.1371/journal.pone.0030095 (PMC3260199; doi:10.1371/journal.pone.0030095)

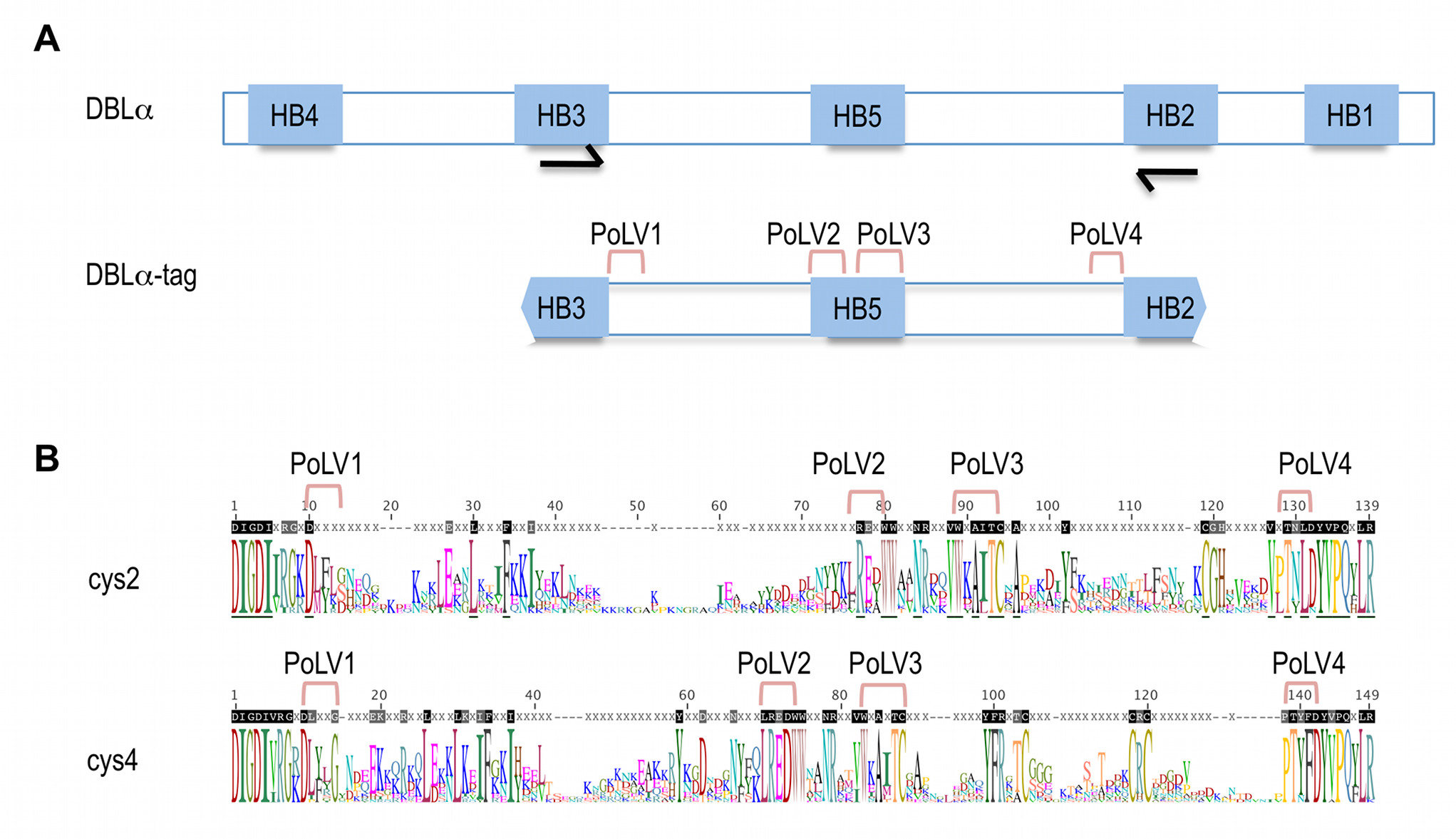

Supplement: Figure S1 — Schematic Overview of the DBLα domain organisation. (A) Indicated is the relative position of homology blocks (HB) 1–5 common to all DBL domains. Universal primers amplifying the DBLα-tag target conserved sequences in HB3 and HB2 and are indicated by black arrows. The “Positions of Limited Variation” (PoLV) 1 to 4, which together with the number of cysteine's in each DBLα-tag form the basis of the DBLα-tag classification. (B) Sequence signature of cys2 and cys4 DBLα-tags. PoLV1-4 are indicated by blue brackets. (TIF) [file pone.0030095.s001.tif]
