# Supplementary material for: T-Cell Responses to the DBLα-Tag, a Short Semi-Conserved Region of the Plasmodium falciparum Membrane Erythrocyte Protein 1
Source: PLoS One. 2012 Jan 17;7(1):e30095. doi: 10.1371/journal.pone.0030095 (PMC3260199; doi:10.1371/journal.pone.0030095)
Supplement: Table S1 — *MUSCLE alignment, shown is the percentage and in parenthesis the number of identical amino acids. (DOC) [file pone.0030095.s002.doc]

**SUPPLEMENTARY INFORMATION**

**Table S1: Comparison of homologous and heterologous DBLα-tags**

| **homologous DBLα-tag** | | | | **heterologous DBLα-tag** | | | |  |
| --- | --- | --- | --- | --- | --- | --- | --- | --- |
| **id.** | **acc. no.** | **cys2** | **group A** | **id.** | **acc. no.** | **cys2** | **group A** | **pairwise identity*** |
| 8477 | FR874897 | yes | yes | 8383 | FR874895 | yes | no | 39.5% (47) |
| 8472 | FR874896 | no | no | 8477 | FR874897 | yes | yes | 40.9% (52) |
| 8472 | FR874896 | no | no | 8383 | FR874895 | yes | no | 37.8% (48) |
| 8585 | FR874899 | no | no | 8618 | FR874900 | no | no | 56.7% (72) |
| 8618 | FR874900 | no | no | 8706 | HE611335 | no | no | 53.2% (80) |
| 8706 | HE611335 | no | no | 8585 | FR874899 | no | no | 50% (76) |
| 6398 | FR874862 | no | no | 6408 | FR874863 | yes | no | 41.9% (52) |
| 6408 | FR874863 | yes | no | 6429 | FR874863 | yes | yes | 40.3% (48) |
| 6429 | FR874863 | yes | yes | 6430 | FR874863 | no | no | 42.6% (55) |
| 6429 | FR874863 | yes | yes | 6433 | FR874863 | no | no | 44.9% (53) |
| 6430 | FR874863 | no | no | 6433 | FR874863 | no | no | 49.2% (64) |
| 6433 | FR874863 | no | no | 6398 | FR874862 | no | no | 47.7% (61) |
| 6433 | FR874863 | no | no | 6408 | FR874863 | yes | no | 40.2% (47) |
| 6485 | FR874863 | no | no | 8349 | FR874863 | no | no | 43.3% (61) |
| 7116 | FR874872 | no | no | 7183 | FR874876 | no | no | 52.3% (67) |
| 7183 | FR874876 | no | no | 7799 | FR874889 | no | no | 50% (66) |
| 7337 | FR874882 | yes | no | 7781 | FR874888 | no | no | 37.1% (49) |
| 7781 | FR874888 | no | no | 8204 | FR874892 | no | no | 46% (64) |
| 7799 | FR874889 | no | no | 7116 | FR874872 | no | no | 43.4 (62) |
| 8204 | FR874892 | yes | no | 6387 | FR874861 | no | no | 43.4 (56) |
| 8204 | FR874892 | yes | no | 7337 | FR874882 | yes | no | 33.6 (43) |
| 8344 | FR874893 | yes | no | 7860 | FR874890 | no | no | 38.6 (49) |
| 8383 | FR874895 | yes | no | 8472 | FR874896 | no | no | 37.8 (48) |
| 8383 | FR874896 | yes | no | 8477 | FR874897 | yes | yes | 39.5 (47) |
| 8482 | FR874898 | no | no | 6485 | FR874867 | no | no | 51.5 (72) |
